# DeID – a data sharing tool for neuroimaging studies

**DOI:** 10.3389/fnins.2015.00325

**Published:** 2015-09-22

**Authors:** Xuebo Song, James Wang, Anlin Wang, Qingping Meng, Christian Prescott, Loretta Tsu, Mark A. Eckert

**Affiliations:** ^1^School of Computing, Clemson UniversityClemson, SC, USA; ^2^Department of Otolaryngology – Head and Neck Surgery, Medical University of South CarolinaCharleston, SC, USA

**Keywords:** de-identification, data sharing, neuroimaging, data anonymization, data auditing

## Abstract

Funding institutions and researchers increasingly expect that data will be shared to increase scientific integrity and provide other scientists with the opportunity to use the data with novel methods that may advance understanding in a particular field of study. In practice, sharing human subject data can be complicated because data must be de-identified prior to sharing. Moreover, integrating varied data types collected in a study can be challenging and time consuming. For example, sharing data from structural imaging studies of a complex disorder requires the integration of imaging, demographic and/or behavioral data in a way that no subject identifiers are included in the de-identified dataset and with new subject labels or identification values that cannot be tracked back to the original ones. We have developed a Java program that users can use to remove identifying information in neuroimaging datasets, while still maintaining the association among different data types from the same subject for further studies. This software provides a series of user interaction wizards to allow users to select data variables to be de-identified, implements functions for auditing and validation of de-identified data, and enables the user to share the de-identified data in a single compressed package through various communication protocols, such as FTPS and SFTP. DeID runs with Windows, Linux, and Mac operating systems and its open architecture allows it to be easily adapted to support a broader array of data types, with the goal of facilitating data sharing. DeID can be obtained at http://www.nitrc.org/projects/deid.

## Introduction

Neuroimaging technologies provide a tremendous opportunity to better understand the healthy and impaired human brain (Schmahmann et al., 1999; Irani et al., 2007). These expensive studies generate voluminous datasets that can be valuable beyond their initial uses (Drevets, 2001). Funding agencies have established guidelines for sharing these data so that they can be leveraged by other scientists and published findings from the data can be replicated by other research groups (Tenopir et al., 2011). For example, the NIH policy on data sharing states “*We believe that data sharing is essential for expedited translation of research results into knowledge, products, and procedures to improve human health*.” and “*The NIH expects and supports the timely release and sharing of final research data from NIH-supported studies for use by other researchers*” (NOT-OD-03-032). Data sharing is thus a significant consideration for researchers. In practice, however, data sharing is time-consuming and complicated when data were collected from human subjects. Methods to share data more easily are necessary to help scientists meet data sharing expectations.

Providing open access data from human subject studies requires that the data do not include any subject identifiers (Van Horn et al., 2004). In the United States, the only legally appropriate mechanism for sharing data according to the Health Insurance Privacy and Portability Act (HIPAA) is to create a limited dataset that excludes identifying information, unless participants have consented that the data can be shared openly. Thus, removal of identifying information from a dataset is necessary and the data should receive new labels that are unlinked to the original data in order to share privacy-protected data. Significant resources are therefore required to prepare a dataset for sharing, which can create a barrier to data sharing, particularly when there are different data types (e.g., images and demographic and/or behavioral data files) that require common labels to link subject data across data types.

The sharing of multiple types of data and numerous variables increases the risk for re-identification due to “direct attacks.” Unique combinations of quasi-identifiers (gender, age, postal code) can often be coupled with public information to re-identify a subject. Caretakers of experimental human subject data must therefore consider the likelihood of re-identification. This risk can be managed by variable exclusion, generalizing data points, or by perturbing clinical data (El Emam et al., 2006). Thus, tools for data sharing should have the ability to perform data generalization when necessary.

Data from structural neuroimaging studies introduces an additional data sharing concern. Structural images of the brain typically include voxels representing the face that can be rendered to visualize the face. There is evidence that rendered faces can be identified at above chance rates when subjects were given pictures to match with the rendered faces (Prior et al., 2009). Thus, voxels representing the face should be removed to reduce the likelihood of re-identification.

Large imaging datasets raises a broader concern that hidden or difficult to detect identifiers are inadvertently shared. This can be a significant issue when dealing with DICOM, Analyze header, and NIfTI files that contain multiple variable fields that are not always clearly apparent, including variables that contain participant IDs and scan dates (Marcus et al., 2007). Therefore, an effective de-identification tool should be able to visualize the image header information and allow users to inspect and de-identify hidden information.

Here we introduce and discuss the novel features of a newly developed Java program for easily de-identifying demographic and/or behavioral, and neuroimaging data, and sharing the data among collaborators. This software was designed with a focus on ease of use through a series of user interaction wizards to: (1) visualize the data; (2) link various data types; (3) remove potential identifiers and/or generalize data; (4) eliminate voxels representing faces; (5) audit and validate the de-identified data, and (6) package the data for sharing. In particular, DeID substantially limits data sharing effort by automatically mapping image filenames with ID labels in a demographic and/or behavioral data file in order to provide new filenames and IDs. With its rich functions, this software can aid researchers in complying with data sharing policies, such as protecting subject privacy, so that investigators and institutions can appropriately and share the data with limited effort or resources. This software was also designed to be cross-platform compatible so that users don't have to switch from their familiar computing platforms for data de-identification and sharing.

## Materials and methods

### Core technologies

**Java**, a general-purpose computer programming language that can be compiled to bytecode running on any Java virtual machine (JVM) regardless of computer architecture, was used to develop DeID software. Java Swing framework was used to design the user interface, which includes a richer set of widgets than Abstract Window Toolkit (AWT), an earlier framework for Java user interface. Swing provides a native look and feel that emulates the look and feel of several platforms. Unlike AWT components, Swing components are written in pure Java and hence are platform-independent.

**FSL BET** (Brain Extraction Tool; Smith, 2002; Jenkinson et al., 2005) removes non-brain tissue from an image of the whole head. It can also estimate the inner and outer skull surfaces, and outer scalp surface. It is robust and has been tested on thousands of datasets from a wide variety of scanners. BET was used in DeID to remove voxels representing facial features.

**MRIcron** (Rorden et al., 2007) is a widely used cross-platform NIfTI format image viewer. DeID utilized MRIcron to allow users to review images at different orientations to ensure brain images are skull-stripped before sharing.

### DeID

**Figure 1:**
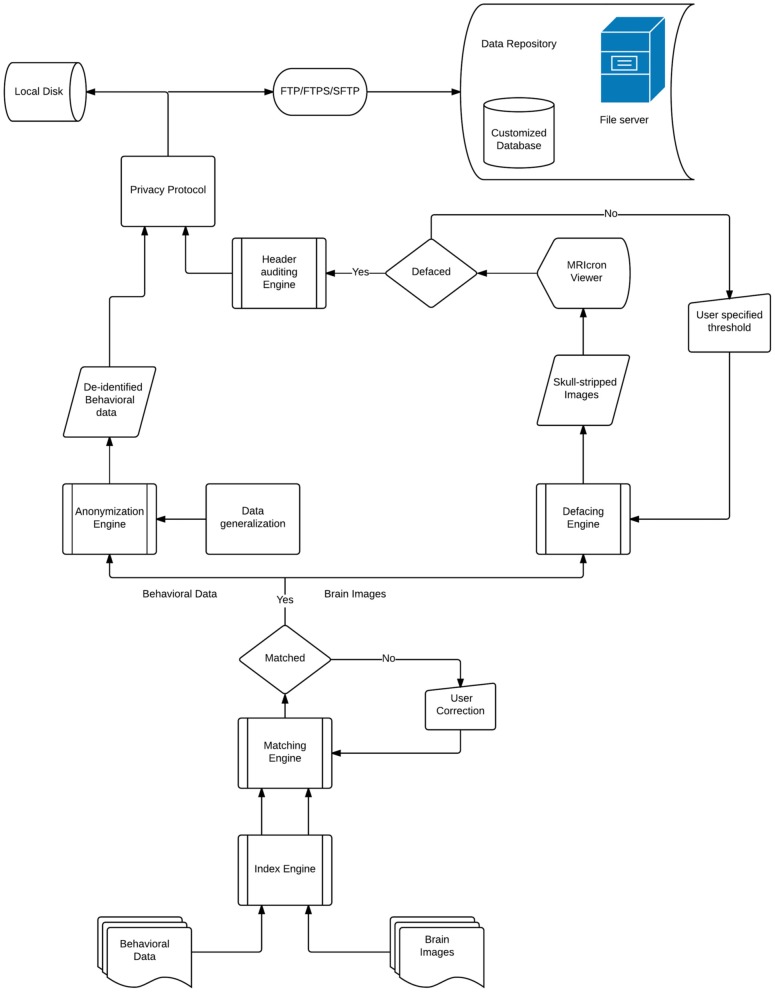
**The architecture of the DeID system**.

DeID makes it possible to share demographic and/or behavioral data, T1-weighted brain images, and can be extended to other datasets. Figure 1 presents a design overview of the tool. These data are first sent through the index engine so that a new and unique ID or label can be assigned to the image file. This value is used by the matching engine to associate the T1-weighed images with an ID variable in a corresponding demographic and/or behavioral data file. The original IDs, which might be tracked back to identifiers, are then removed from the image and data files. The demographic and/or behavioral data then goes through the anonymization engine to be de-identified. The brain images are subsequently skull-stripped using the defacing engine that is essentially a call to BET. The de-identified demographic and/or behavioral data along with the skull-stripped images are then either saved to the user's local disk or sent to the remote data repository. These functions and the data processing flow are also summarized in Figure 1.

There are eight primary functions for data de-identification in the DeID system,

- Data selection: Users select the image files (NIfTI or Analyze format) and a data spread sheet (txt or xls format). Users should consider editing the corresponding spread sheet or generalize the data in order to reduce the likelihood of re-identification by removing unique patterns of variables.
- Associative match: DeID matches filename and/or the directory path of the image files with participant ID values in a data file.
- Variable selection: Users can remove specific variables from the data file (e.g., scan or test date variables).
- Skull stripping: BET skull-stripping removes voxels that represent identifiable facial features.
- Quality control: Provides users views of the skull-stripped brain images along with the original ones, and allows users to re-BET some images with different skull-stripping parameters, if necessary.
- Image rendering: Allows users to view the defaced images in different orientations to ensure that voxels representing the face have been removed.
- Header auditing: Users can view the header file of each image and alter specific fields to remove identifiable information in the header file.
- Data sharing: DeID packages the de-identified data and a log file containing information about the user who prepared the data and the data sharing preferences to send the data in a single data package.

#### Data selection

**Figure 2:**
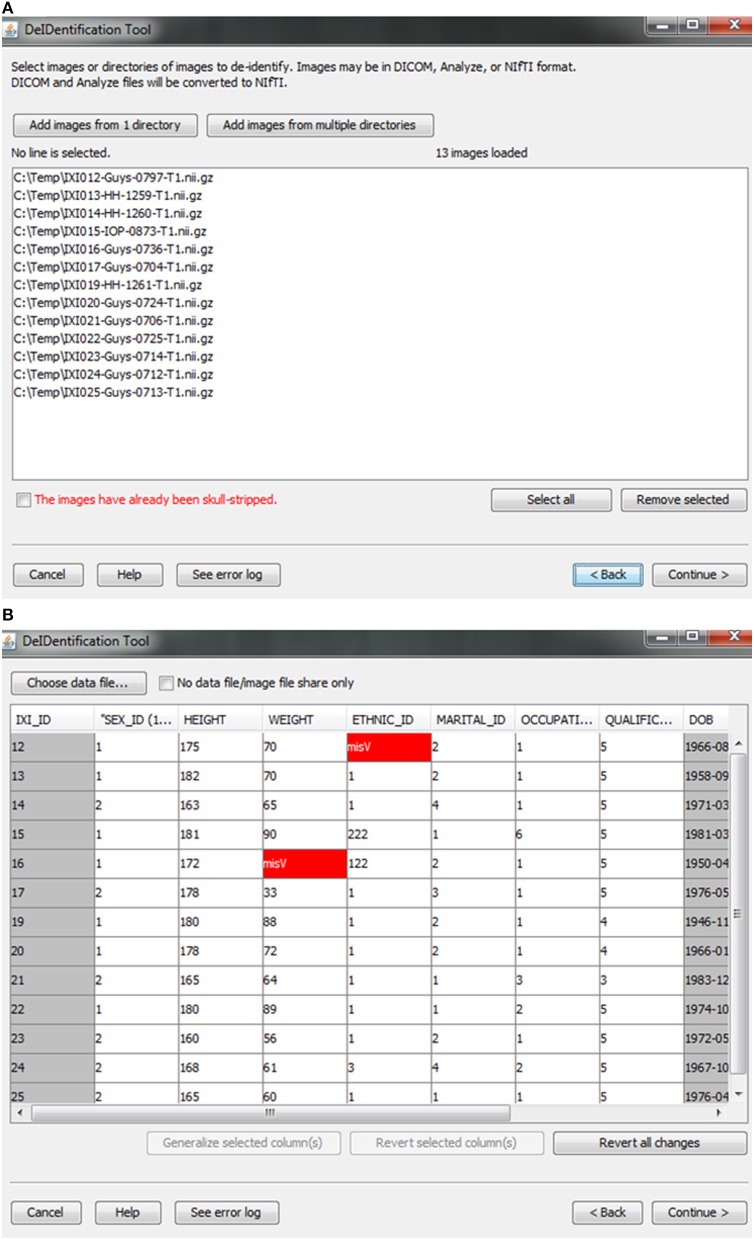
**Data selection**. **(A)** Image files are selected. **(B)** An xls or txt data file is selected. Note that missing values are highlighted in red to inform users about missingness that might be correctable. The highlighted columns such as ID and DOB will be pre-removed in a subsequent step shown in **Figure 5**.

Users are provided with an interface to choose the source data (image and data files) on a local disk, as shown in Figure 2. Analyze 7.5 (hdr/img) and NIfTI (nii) format image files are supported in DeID. These images can include voxels representing the face as these voxels will be removed during the skull-stripping step described below. The option to share neuroimaging data that has already been skull-stripped or that do not require stripping (e.g., 4D diffusion datasets) is also supported to utilize other auditing functions of DeID to meet users' various demands. It is not uncommon that multiple copies of an image are collected for each subject and for this reason DeID was designed to accept multiple images for each subject. All of the images can be selected from a single directory or stored within subject-specific directories.

**Figure 3:**
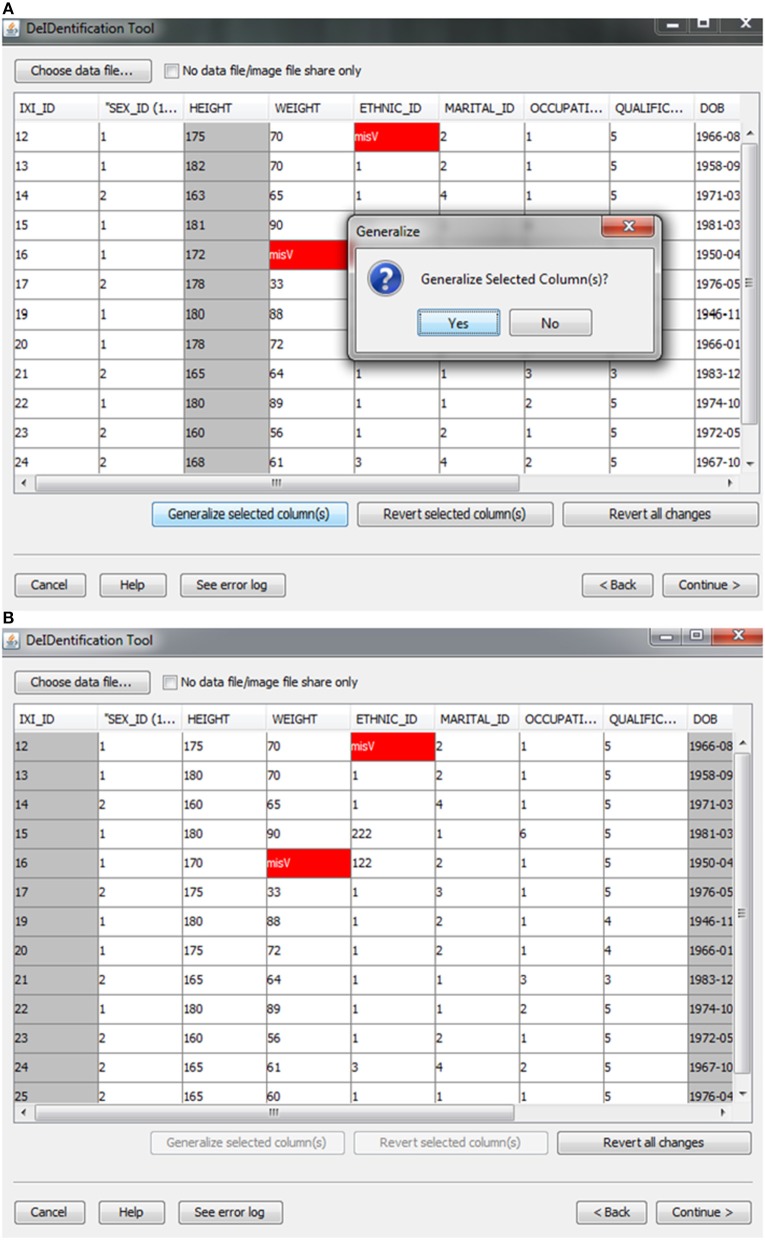
**Data generalization**. Users can select specific column(s) to generalize **(A)**. The result of generalizing the “Height” column is shown in **(B)**.

After the images have been selected, users are prompted to select a corresponding data file that may contain demographic and/or behavioral data. Even after explicitly removing identifying information such as name and date of birth, it is still possible to link released records back to their identities by extreme values (e.g., a 96 year old), as well as matching some combination of non-identifying attributes such as sex or zip code. Data randomization (anonymization) and generalization approaches have been proposed to mitigate risk (Wang et al., 2004; Bayardo and Agrawal, 2005; Fung et al., 2007; El Emam et al., 2009). DeID provides a data generalization function to smooth values by rounding quantitative data and reduce the possibility of re-linking the corresponding data to the subject. As shown in Figure 3, data generalization of the “HEIGHT” column smoothed values while preserving the data structure. In addition, the data selection interface allows users to identify missing data and edit the cells if the data is available. A “Revert changes” button is provided to prevent inadvertent operations.

#### Associative match

**Figure 4:**
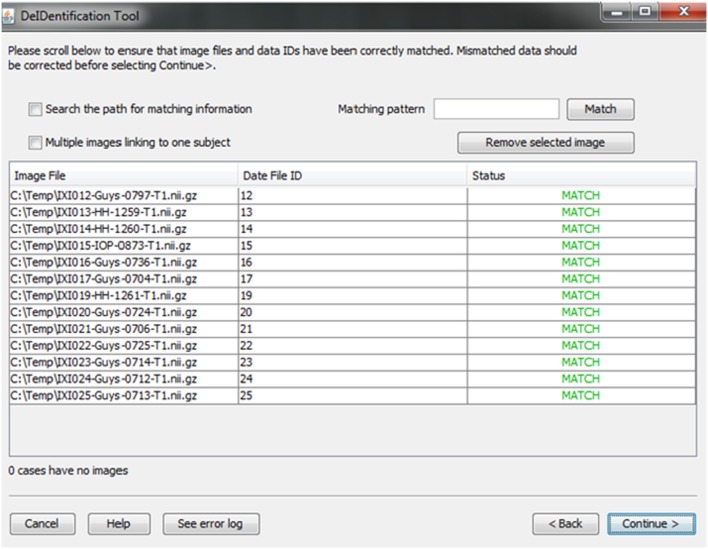
**Associative match showing matching image filename and ID values**. A mismatch can be corrected manually by editing the cells or the user can select options to search the path for matching values, correct mismatches that are due to multiple images for a single subject, or provide a matching string pattern.

A new and unique ID is assigned to each subject in the data file and the subject's corresponding image(s). This step unlinks the ID value to any personal health identifiers in the contributor's records that can typically be tracked using the original ID. Once the image and data files are selected, the system will link them according to the common unique ID that appears in both image file names and the first column in the spreadsheet. This step connects the images and associated variable values in the data file (Figure 4). This step also helps users to verify that the image files and variable values are correctly matched. The status column will display MISMATCH when an item is not matched (Figure 4). A mismatch can occur because an image is missing for a case in a data file, there are multiple images for each subject, or because the filename is not an exact match for the ID label in the data file. The latter two conditions are dealt with by selecting a box indicating that multiple files are present for each subject and by searching the path for the matching ID label or by specifying a wildcard pattern. Users can select a missing value option that will fill the data file with a missing data code for the former case in which an image is present for a case with missing data in the data file.

#### Variable selection

**Figure 5:**
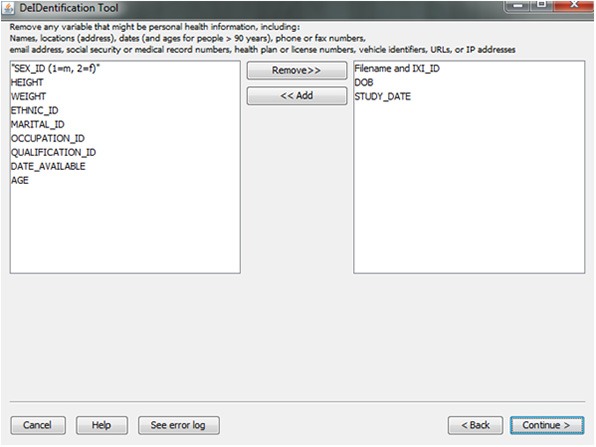
**Variable selection**.

**Figure 6:**
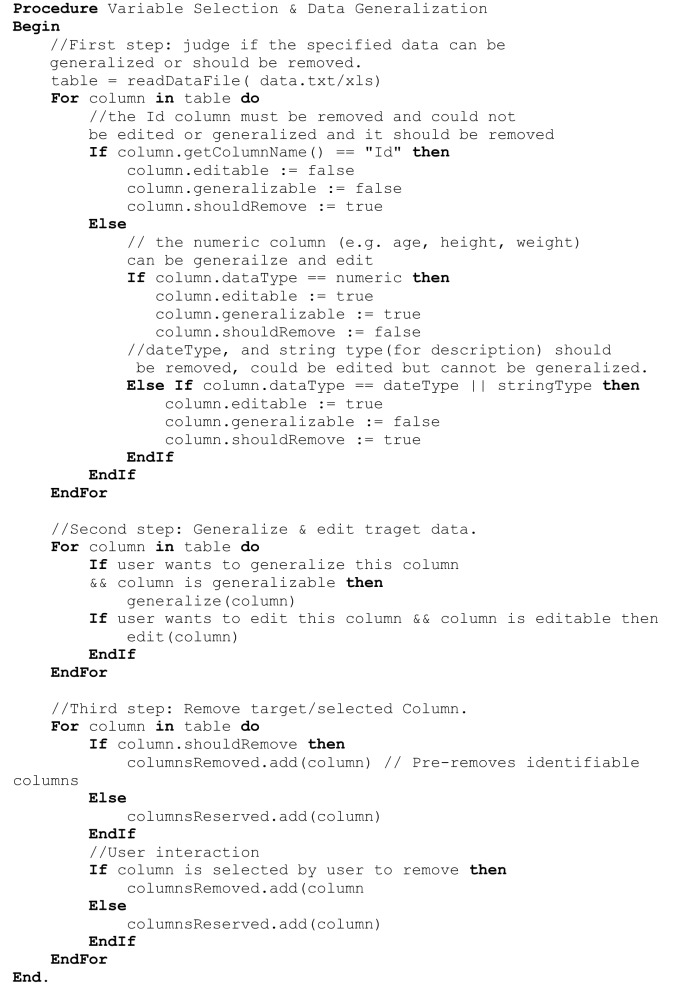
**Algorithms associated with data generalization and variable selection**.

In the course of preparing a data file for sharing, particularly when there is a large number of variables in a spreadsheet, subject identifiers may be inadvertently included in the data file. Users are prompted to view the variable names at the top of their data file and have the option to remove variables such as names, addresses, test dates that should be removed from the dataset. Figure 5 shows this interface, which includes guidance about what identifiers to remove. In addition, DeID automatically detects and pre-removes identifers such as date of birth, dates, and names to help users reduce the time involved with this procedure. Users have the ability to easily reverse this process if specific columns need to be shared. The algorithms involved in demographic and/or behavioral data de-identification are illustrated in Figure 6.

#### Skull stripping and image rendering

**Figure 7:**
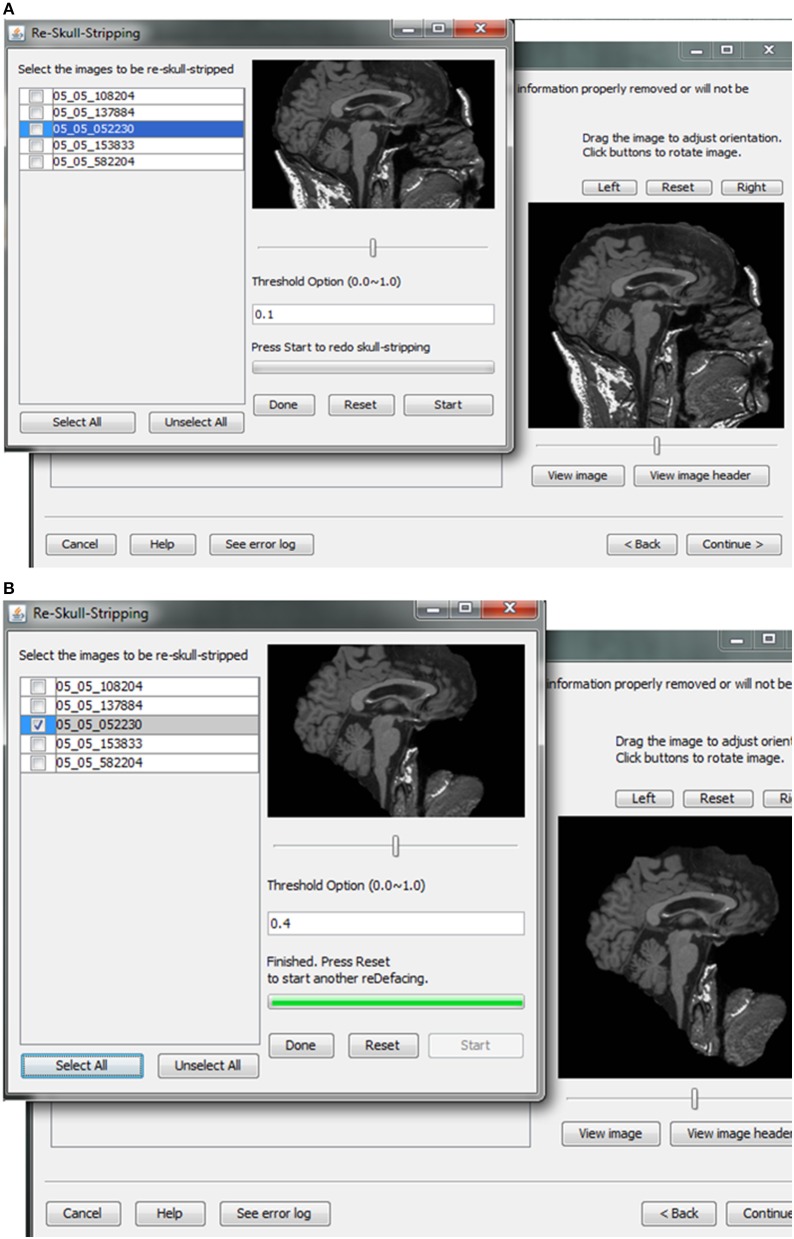
**Skull stripping**. If the images were not appropriately skull-stripped, as shown in **(A)**, users can choose to re-skull-strip specifying a different threshold **(B)**.

Skull-stripping is performed to remove voxels representing the face. Tools such as ROBEX (Iglesias et al., 2011), mri_watershed (Ségonne et al., 2004), and mri_deface (Bischoff-Grethe et al., 2007) are excellent tools for removing voxels that represent the face, but we chose to use BET (Smith, 2002) for multiple reasons. BET is flexible in handling mulitple image orientations, easy to use for naïve users, computationally efficient, and could be implemented across operating systems. A small tradeoff for these benefits is that voxels representing much of the neck and skull might remain in the skull-stripped image, as shown in Figure 7. Skull-stripping algorithms such as BET estimate the brain outline within a range by providing a fractional intensity threshold parameter (0–1) with the default value being 0.5. This threshold can be varied if too few voxels representing the face are removed (Figure 6) or too many voxels are removed, including those representing the brain.

**Figure 8:**
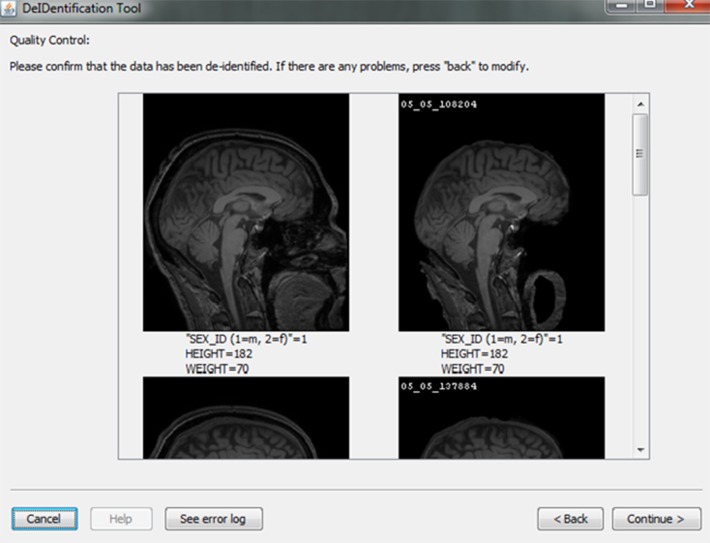
**Quality control presenting skull-stripped images side by side with the original ones**. The corresponding demographic and/or behavioral data are shown to ensure the data integrity.

After skull-stripping, the 2D slices of each image can be inspected to evaluate the extent to which BET removed voxels representing the face and brain. Users can view 2D images sliced from the 3D image data from different positions (slice the bar) or orientations (click on the image). MRIcron can also be called to render the images so that the user can directly inspect whether the face is still visible after skull-stripping. A montage function provides a third visualization method to allow the user to scroll through all of the skull-stripped images as shown in Figure 8. In addition to image inspection, this montage function also enables the user to spot check the data to ensure that the images and variable values are correct relative to the original data. This additional auditing function is included to ensure the integrity of the data.

#### Header auditing

**Figure 9:**
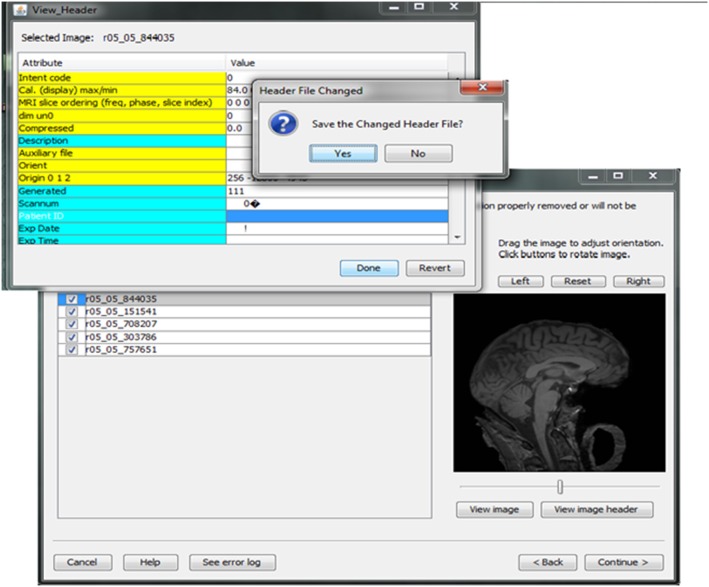
**Header visualization and auditing**.

Contributors may be uncertain that all of the potential identifiers have been detected and removed from a header file given the numerous fields in these files. For example, in Analyze 7.5 (hdr/img) images, the fields “Description,” “Scan number,” “Patient ID,” “Experiment Date,” could play a significant role in re-identifying the subject or be used to confirm the subject's identity from large databases of neuroimaging data. This kind of hidden information is difficult to detect using many existing tools because of visualization and editing limitations. DeID provides a header visualization function that enables users to audit image headers. As shown in Figure 9, potential fields such as “Description” are highlighted differently from other fields to indicate that they can be edited, while fields such as “Dimensions” cannot be edited to preserve image format and display parameters that are necessary for subsequent image processing.

#### Data sharing

**Figure 10:**
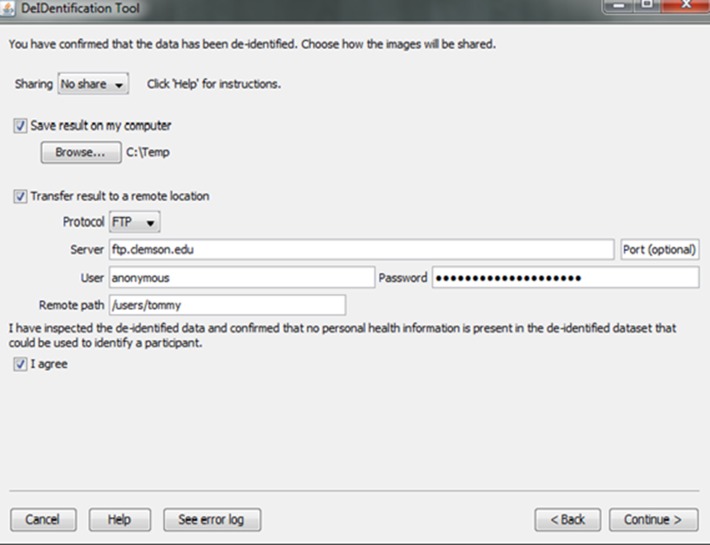
**Data sharing**.

**Figure 11:**
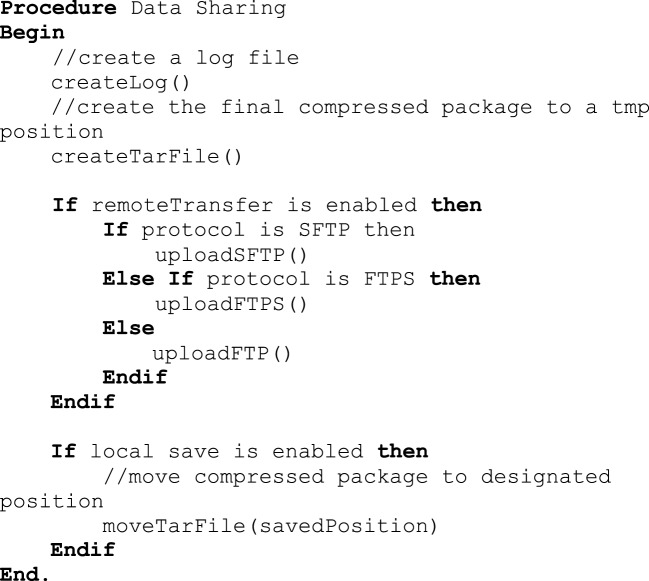
**Data sharing procedure**.

Investigators and institutions sharing data can be concerned about how the data will be accessed by other users. For this reason, users specify whether the data can be: (1) shared in an open access format; (2) shared with limited access in a data enclave or secure computing environment; or (3) shared only with the investigator(s) receiving the data from the contributor. This information is included in a log file with the contributor's name, institution, and date that DeID was used. Once the user indicates that the data has been inspected to ensure there are no personal health identifiers, the data is packaged as a tar.gz file and either stored on the local machine or transferred to a recipient's server using an ftp/ftps/sftp protocol (Figure 10). The process of data sharing is shown as pseudo code in Figure 11.

### Challenges

#### Image format compatibility

DeID supports Analyze 7.5 and NIfTI format. NIfTI files can exists in two forms, a single nii file or two separate hdr and img files. While Analyze 7.5 images also come with hdr/img format, DeID auto-detects the image format to differentiate Analyze and NIfTI formats by examining the last four bytes of the header file excluding the extension fields. NIfTI files with hdr/img pairs are identified by an “ni1” value. Nii files are identified by the four bytes “n + 1.” Analyze 7.5 files are identified based on the absence of the “ni1” and “n + 1” information.

#### Cross platform

**Figure 12:**
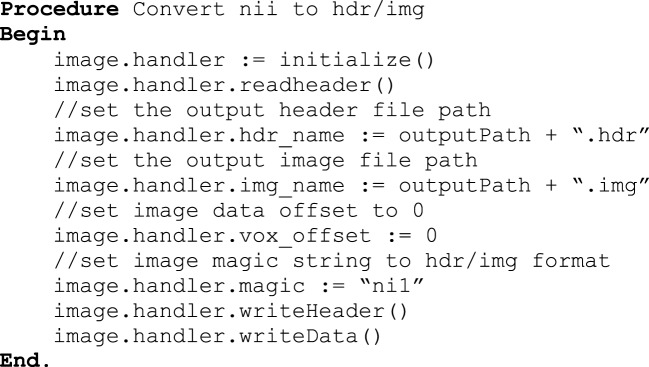
**nii to hdr/img conversion**.

Windows has almost 90% of the operating system market share and for that reason it was essential to design a Windows compatible version of DeID. One difference in the development of DeID for Windows compared to Linux and Mac versions is that DeID for Windows calls BET using the MRICron implementation of BET because it is the only implementation of BET for Windows. Importantly, this Windows implementation of BET only supports the hdr/img file bundle. DeID therefore converts nii files to hdr/img pairs to in order to perform skull-stripping. Figure 12 presents the key procedures of this conversion function.

Another concern when dealing with cross-platform compatibility is software package dependencies. Even though Java is designed to be cross-platform compatible, it requires necessary libraries or run-time environments installed on different platforms. Furthermore, different dependent library packages are required under different platforms. It is a great challenge for users to manually install all required dependency packages especially when there are recursive dependencies. DeID integrates all the dependencies in a single package so that any user can run DeID directly without worrying about installing any packages.

#### Memory constraint

**Figure 13:**
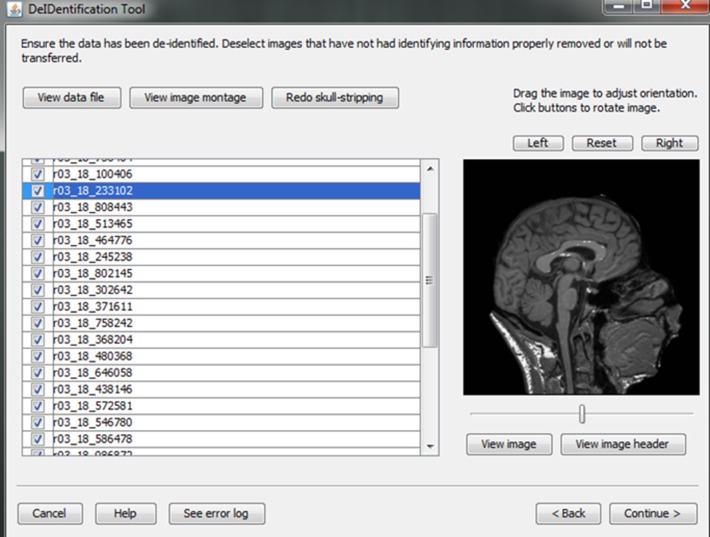
**Image rendering**.

There are potential memory limitations to using DeID because of the large size of T1-weighted images (e.g., 20 MB for a 256 × 256 × 150 16 bit image). To address this issue, DeID adopted a “double-buffering” technique to load images instead of loading all the images into the memory simultaneously. This technique is applied when rendering images (Figure 13) and montage creation (Figure 8).

**Figure 14:**
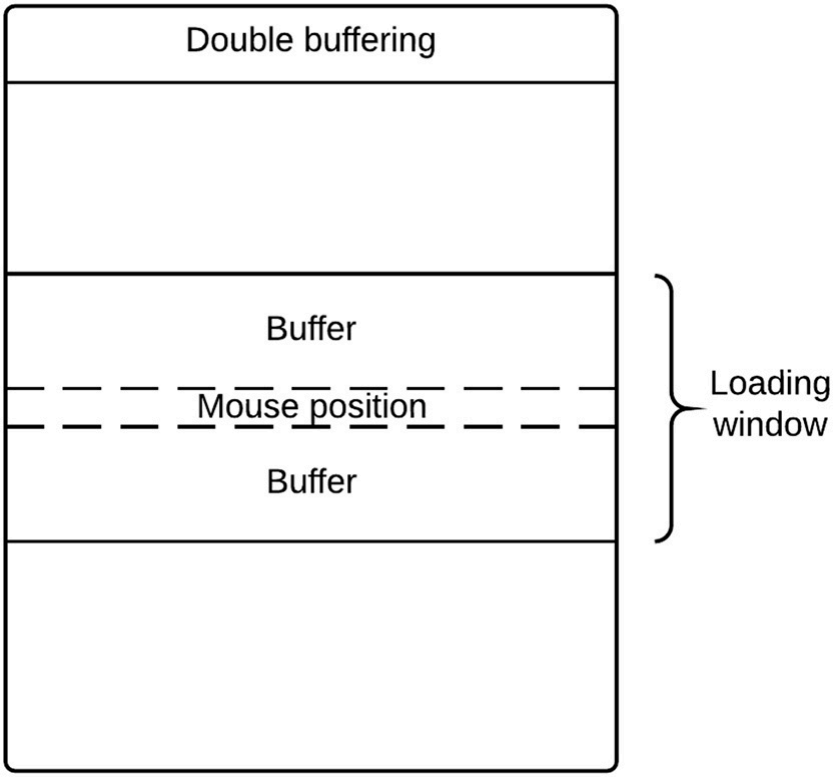
**Double buffering mechanism loading images on-the-fly**.

Loading all images in memory is a waste of processing resources and is potentially problematic for computers with relatively limited memory. DeID captures user mouse movement and only loads images into memory for display when they are about to be shown in the viewing area. Figure 14 highlights the key point of double buffering technique. Only the images within the loading window are loaded into memory while the window size is determined by the current available memory avoiding the risk of memory overflow.

## Results

One of the challenges in data sharing is the efficiency and scalability of de-identification. More often than not, large amounts of structural images along with demographic and/or behavioral data need to be de-identified simultaneously without incurring significant human intervention. This requires the data sharing tool to be robust and able to handle large amounts of data efficiently. DeID was tested against scalability and computational efficiency on a dual core 2.40 GHz CentOS machine with 4 GB memory. Table 1 demonstrates that DeID can sustain reasonable computational efficiency for large numbers of structural images. The time in Table 1 includes the total time involved in index randomization, demographic and/or behavioral data anonymization, brain images skull-stripping along with montage creation. By using the “double buffering” technology, the DeID tool can theoretically handle as many images as necessary.

**Table 1:** **Scalability and computational efficiency view of DeID**.

**No. of images**	**1**	**5**	**25**	**30**	**100**	**200**	**300**	**500**
Time (s)	7	4	272	553	1132	2250	3443	5621

## Evaluation

DeID was tested extensively by the writers of the code using open access data (http://biomedic.doc.ic.ac.uk/brain-development/). Our team of neuroimaging scientists also evaluated DeID with data collected for aging and pediatric studies. This testing was not limited to but included the following key functions: (1) data import and associative matching using datasets with single and multiple images for each case, as well as evaluation of the data import function for imaging data stored with different directory structures (e.g., all images in one directory and images in subject-specific directories); (2) associative image and data matching when there was missing or unusual characters in data files; (3) skull-stripping when images had different acquisition orientations; and (4) display of skull-stripped images and the re-skull-stripping function. We also invited other research groups to perform testing of the software. This testing led to the development of the double-buffering method to avoid memory constraints for quite large datasets.

Automated testing was also performed using the 581 cases from the brain-development.org open access database for the data anonymization and the assignment of new ID labels, a step that is to protecting subject privacy and the integrity of the data. The open access data was used so that we could replicate testing in future versions of DeID and so that other users could perform their own tests with the same open access data. In the data anonymization step, the old ID labels are removed and the data spreadsheet is populated with new ID labels. Automated testing compares the initial uploaded spreadsheet to the final spreadsheet that is de-identified and can be shared with other researchers. The column or variable names and their values are compared between spreadsheets to ensure that all values, with the exception of the new ID labels, are identical. This testing, which is performed 1000 times with random sampling for the 581 cases, reports a failure if any value in corresponding cells does not match. Testing is also performed to ensure that the assignment of new ID labels does not duplicate an existing ID label. Random ID labels are assigned until there is no duplication with the original IDs. This function is built into the DeID software. Again, manual data quality checking is also available to contributors who can verify that images and their associated data have been correctly linked during the associative matching step (Figure 8).

## Discussion

There is a growing expectation that researchers share clinical and experimental data (Poline et al., 2012) with the hope that increased sample sizes and novel methods can lead to more rapid scientific discoveries (Teeters et al., 2008; Miham, 2012; Poldrack, 2012) and enhance scientific integrity. We have provided the DeID tool to increase the feasibility of data sharing. Importantly, we designed DeID to limit the risk of unauthorized sharing of personal health information and to ensure that researchers consider privacy issues when they share data.

Many researchers do not plan for data sharing when designing their IRB protocols and consent forms, despite mandates to share data from funding agencies. This can be advantageous for neuroimaging studies because requesting authorization can produce sampling bias (Harris et al., 2008), but this also means that most datasets do not have subject approval for sharing. Therefore, the legally appropriate mechanism for sharing data according to the HIPAA is to create a limited dataset that excludes identifying information. Significant effort is required to de-identify data (Poldrack et al., 2013; Haselgrove et al., 2014). The simple wizard design of DeID mitigates this effort and facilitates data sharing. In addition, auditing functions in DeID provide users confidence that multiple datasets have been properly linked for each participant. These functions aid in the detection of remaining identifying information, such as remaining voxels representing a face.

The risk of re-identification is a significant privacy concern even with the removal of personal health information. The auditing strategy in DeID (data generalization, variable selection, quality control, header file auditing and image rendering) serves to limit this risk, which is ultimately influenced by the clinical or experimental nature of the data (i.e., less risk for experimental studies of questions broadly relevant to a population). While we designed DeID to help researchers limit the risk of privacy violations, it is ultimately the responsibility of the parties sharing and receiving the data to ensure that the data has been de-identified. It is for this reason that DeID includes a required step for users to indicate that the data have been inspected and have been de-identified. This information is logged and stored with information about who processed the data and when. The result is an audit trail to better understand when and by whom a de-identification error may have occurred. Ideally, DeID will help to prevent privacy violations and make it easier for research groups to share data.

Again, DeID ensures that data are de-identified by removing any link between the original ID and the new random ID labels. One important consideration for users of DeID, and researchers sharing data in general, is that incidental findings may be found in shared data. There has and continues to be variation in how researchers and Institutional Review Boards prefer to deal with screening and sharing incidental findings (Nelson, 2008; Borra and Sorensen, 2011). Thus, the removal of all identifying information raises a question about what to do when incidental findings are observed in shared neuroimaging data. Our recommendation is to share every incidental finding with a contributor by providing images of the finding and any accompanying information about the case that was shared with the MRI scan. This approach will help the contributor identify the case in their dataset so that the finding can be communicated and so that clinical decisions can be made, while ensuring that the shared data remains de-identified. We encourage researchers to consider this incidental finding issue, but not to let it deter data sharing that has the potential to significantly enhance advance research and increase scientific integrity.

Finally, we also encourage researchers to develop their own functions within DeID. The software has a GNU Library General Public License (LGPL) so that users are free to modify DeID, while associated libraries having their own licensing cannot be modified. DeID can be obtained at http://www.nitrc.org/projects/deid.

## Funding

This work was supported by 5R01HD069374. This investigation was conducted in a facility constructed with support from Research Facilities Improvement Program (C06 RR14516) from the National Center for Research Resources, National Institutes of Health.

### Conflict of interest statement

The authors declare that the research was conducted in the absence of any commercial or financial relationships that could be construed as a potential conflict of interest.
